# New Approach for Risk Estimation Algorithms of *BRCA1/2* Negativeness Detection with Modelling Supervised Machine Learning Techniques

**DOI:** 10.1155/2020/8594090

**Published:** 2020-12-09

**Authors:** Hulya Yazici, Demet Akdeniz Odemis, Dogukan Aksu, Ozge Sukruoglu Erdogan, Seref Bugra Tuncer, Mukaddes Avsar, Seda Kilic, Gozde Kuru Turkcan, Betul Celik, Muhammed Ali Aydin

**Affiliations:** ^1^Istanbul University, Oncology Institute, Department of Basic Oncology, Division of Cancer Genetics, 34093 Fatih, Istanbul, Turkey; ^2^Istanbul University-Cerrahpasa, Engineering Faculty, Computer Engineering Department, 34320 Avcilar, Istanbul, Turkey

## Abstract

*BRCA1/2* gene testing is a difficult, expensive, and time-consuming test which requires excessive work load. The identification of the *BRCA1/2* gene mutations is significantly important in the selection of treatment and the risk of secondary cancer. We aimed to develop an algorithm considering all the clinical, demographic, and genetic features of patients for identifying the *BRCA1/2* negativity in the present study. An experimental dataset was created with the collection of the all clinical, demographic, and genetic features of breast cancer patients for 20 years. This dataset consisted of 125 features of 2070 high-risk breast cancer patients. All data were numeralized and normalized for detection of the *BRCA1/2* negativity in the machine learning algorithm. The performance of the algorithm was identified by studying the machine learning model with the test data. *k* nearest neighbours (KNN) and decision tree (DT) accuracy rates of 9 features involving Dataset 2 were found to be the most effective. The removal of the unnecessary data in the dataset by reducing the number of features was shown to increase the accuracy rate of algorithm compared with the DT. *BRCA1/2* negativity was identified without performing the *BRCA1/2* gene test with 92.88% accuracy within minutes in high-risk breast cancer patients with this algorithm, and the test associated result waiting stress, time, and money loss were prevented. That algorithm is suggested be useful in fast performing of the treatment plans of patients and accurately in addition to speeding up the clinical practice.

## 1. Introduction

Machine learning is a computer-based predictive method or an estimation algorithm which makes an assumption of a hypothesis and uses this assumption for the estimation of the unknown condition using various mathematical and statistical methods. Thanks to this association between mathematics and computer science, the difficulties on creating and calculating the statistical models from large datasets including billions of data are resolved [1]. Machine learning is accepted as a branch of artificial intelligence owing to obtaining significant patterns from the samples such as human intelligence. Recently, machines were found to learn the duties that are regarded highly complex, and the computer-aided diagnostic and decision support systems of machine learning algorithms were demonstrated to be ultimately useful. After discovering this, the question of how machine learning will enter in the practice particularly in medicine comes to mind. Up to present, various biological mechanisms were revealed using the conventional statistical modelling and analysis methods [2–8]. The biological and medical data produced in the area of biology and medicine have become more heterogeneous and complex with the rapid development of highly productive technologies in recent years, and the evaluation of these data becomes highly difficult with the known statistical analyses. More different methods such as advanced machine learning algorithms are required considering this rapid development in biology and medicine and with the increase of the size of these data. Therefore, there is a need for the development of more effective and productive approaches such as advanced machine learning algorithms and network-based analyses for analysing such tremendous data. The investigation of the literature studies showed that machine learning is successfully practiced in resolving numerous problems such as classification, regression, and clustering [9, 10].

The studies on the use of machine learning algorithms in medicine are particularly based on the evaluations associated with the medical screening systems [11, 12]. These algorithms are used for various difficult tasks such as pulmonary embolism segmentation [13, 14] by performing angiography with computed tomography (BT), detection of polyp in colon cancer using virtual colonoscopy or CT [15, 16], detection of breast cancer diagnosis using mammography [17], brain tumor segmentation using magnetic resonance imaging (MRI) [18], and detection of the cognitive condition of brain (e.g., Alzheimer's disease) [19–21] using functional MR imaging.

The cause of the breast cancer is still unknown, and there is no available efficient prevention method against breast cancer. Therefore, early diagnosis or the identification of the present risk are the most important steps in treatment, and in the prevention of breast cancer [22]. Important steps have been put forward with the developments of effective diagnostic techniques and treatment methodologies in breast cancer in recent years [23]. Breast cancer results in the death of many people each year and constitutes a significant health problem worldwide. Breast cancer is the second most prevalent cancer after lung cancer and is the main reason of mortality in women worldwide [24]. In accordance with the Globocan 2018 data, the most prevalent cancer among women and men with a rate of 10.6% was breast cancer after lung cancer [25]. Approximately 80% of hereditary breast cancer cases develop due to the mutations on *BRCA1/2* genes [26]. *BRCA1/2* genes are tumor suppressing genes and have a role in response to cellular stress through the activation of the DNA repair processes. The pathogenic mutations in *BRCA1/2* genes show that breast cancer has hereditary characteristic and increases the risk of getting breast cancer diagnosis [27]. Although approximately 12% of women in general population develop breast cancer all through their life, 72% of women with pathogenic *BRCA1* mutation and 69% of women with pathogenic *BRCA2* mutation are diagnosed with breast cancer at a stage of life. In addition, researchers reported that approximately 40% of women with pathogenic *BRCA1* mutation and approximately 26% of women with pathogenic *BRCA2* mutation develop secondary breast cancer in the contralateral 20 years after the first diagnosis of breast cancer [28]. Therefore, the identification of the *BRCA1/2* gene mutations, the fast practice, and concluding of the gene analysis are highly important in planning the treatment and identifying the secondary cancer risk. The completion of the study of this test which is difficult, expensive, and long-lasting and therefore stressful for patients is significantly important in the rapid selection of the treatment protocols and rapid change of treatment when required.

There is no algorithm which identifies the *BRCA1/2* negativity in the literature; in addition, there is no available algorithm that describes the *BRCA1/2* negativity by only evaluating the clinical and demographic data of patients without performing the *BRCA1/2* gene test in high-risk breast cancer patients with a family history. There is a need for algorithms which identify the *BRCA1/2* gene mutation negativity in a short time and enabling the planning treatment rapidly by reducing the health costs and workload of cancer genetics polyclinic and laboratory. Therefore, an algorithm which identifies the individuals with negative *BRCA1/2* gene mutation in short time was developed in the study by considering all the clinical, demographic, and genetic features of the total of 2070 patients.

## 2. Materials and Methods

**Figure 1:**
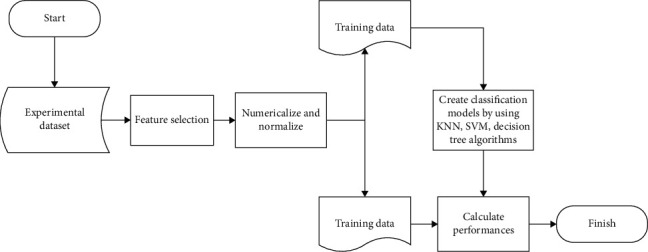
Flowchart of the proposed method.

The study flow was arranged as follows in the study conducted to develop an algorithm in order to determine the negative cases for *BRCA1/2* mutation without having a *BRCA1/2* gene test. The experimental dataset with 2070 individuals were created and pre-processing step was started with the feature selection. Datasets obtained in the feature selection were independently performed in pre-processing procedure, and were normalized and numeralized for developing classification models. The training and testing dataset were generated for the supervised machine learning models for detection of the *BRCA1/2* negativity. All the work flow diagram for machine learning algorithm is presented in Figure 1 and all steps are detailed below.

### 2.1. Creation of Experimental Dataset

A dataset, including all the clinical, demographic, and genetic features of 2070 breast cancer patients who presented to the Cancer Genetics polyclinic in the Cancer Genetics Division of the Oncology Institute in Istanbul University for *BRCA1/2* genetic testing between 1999 and 2019, was created. Some part of this raw data is presented in Table 1. The study was approved by the Local and Clinical research Ethics Committee of Istanbul University according to the tenets of the Declaration of Helsinki. All patients were informed about the study, and the consents were granted in the scope of the cancer genetics polyclinic. The clinical parameters which were particularly important for breast cancer were identified in the study. These parameters were classified as the modifiable and nonmodifiable parameters. A data classification technique which might predict the hereditary breast cancer was used with the use of a function that included the modifiable and nonmodifiable factors in the study. The selected nonmodifiable factors were menopause, the result of the *BRCA1/2* genes, family history, and receptor condition; however, the modifiable factors were the use of oral contraceptive, alcohol consumption, hormone therapy, breast feeding, and smoking.

### 2.2. Preprocessing of Dataset: Feature Selection

Feature selection is a method which may be used for removing the unnecessary, irrelevant, and invalid features which have no contribution to the accuracy of a predictive model. The raw dataset of 2070 high-risk breast cancer patients included 125 modifiable and unmodifiable parameters. First, the raw dataset was performed feature selection because there were numerous variables which did not affect the *BRCA1/2* negativity or there were unnecessary, irrelevant, and invalid features in the dataset. Then, the raw dataset consisting of 125 features was evaluated using Pearson's correlation method using the Statistical Package for the Social Sciences (SPSS) v.21, and statistically significant (*p* < 0.05) features were selected. After the feature selection stage, two different datasets were generated for 1460 and 1532 breast cancer patients and their 26 and 9 variables. The patient and feature data of the datasets are given in Table 2.

**Figure 2:**
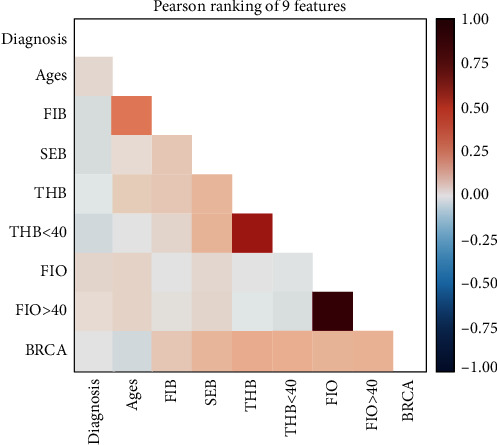
Association between the features. Dia; diagnosis; FIB; number of first degree relative in breast; SEB; number of second degree relative in breast; THB; number of third degree relative in breast; THB<40: number of third degree relative in breast under age of 40; FIO: number of first degree relative in ovarian; FIO>40: number of first degree relative in ovarian above age of 40.

After Pearson's correlation analysis, the statistically most significant 9 features and their associations in the raw dataset are given in Figure 2. As shown in Figure 2, 0.75-1 was detected between FIO and FIO > 40, however, 0.50-0.75 between THB and THB < 40, 0.25-0.50 between the age and FIB, and -0.25-0.25 between other 5 variables.

### 2.3. Normalization and Numeralization of Dataset

Two different datasets obtained in the feature selection procedure were independently performed in a preprocessing procedure and were normalized and numeralized for developing classification models of the dataset for the machine learning algorithms. The nonnumerical data included in the dataset during this procedure were numeralized. The numerical data were degraded between 0 and 1 for normalization.

(1) $$\begin{array}{c} z_{i}=\frac{x_{i}-\min\left(x\right)}{\max\left(x\right)-\min\left(x\right)}. \end{array}$$

*z*_*i*_ indicates the normalized *i* data. *x*_*i*_ is normalized between 0 and 1 interval by division with the value between min(*x*) and max(*x*) after deduction from the minimum *x* value included in the dataset to be normalized.

All data were numeralized and normalized for obtaining more successful results in the machine learning algorithm.

### 2.4. Creation of the Supervised Machine Learning Models

After the preprocessing procedure, the first dataset with 1460 patients and 26 features and the second dataset with 1532 patients and 9 features were, respectively, evaluated using the *k* nearest neighbours (KNN), support vector machines (SVM), and decision tree (DT) machine learning algorithms, and classification models were generated for the detection of the *BRCA1/2* negativity. *k* nearest neighbour is a supervised machine learning technique. *k* performs the classification procedure in accordance with the nearest neighbour. SVM classifies the samples by generating the optimum hypersurface which differentiates the samples in the space. DT is a supervised machine learning technique which performs classification by generating a decision tree.

In the scope of the study, both 2 datasets that were normalized and numeralized were divided into two groups: 67% as the training dataset and 33% as the test dataset. Three different classification models were developed with training of KNN, SVM, and DT algorithms using the training dataset. The performances of the developed classification models were measured using the test data and were comparatively presented.

## 3. Results and Discussion

**Figure 3:**
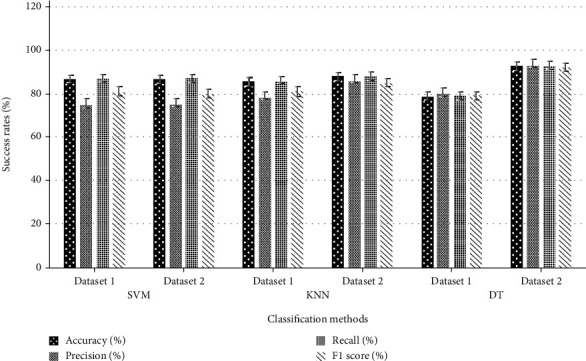
The comparative demonstration of the performances of the algorithms. The *x*-axis shows the classification models for Dataset 1 and Dataset 2. The *y*-axis shows the success rates (%) of the algorithms. SVM: support vector machines; KNN: *k* nearest neighbours; DT: decision tree.

The raw dataset involving 125 features of 2070 high-risk breast cancer patients was used for the detection of the *BRCA1/2* negativity. The number of features was reduced to two different datasets as 26 and 9 using the experimental dataset dimension reduction procedure. All the learning algorithms were separately performed to both 2 datasets, and the success rates were measured. The success rates of algorithms used in developing the classification models for Dataset 1 and Dataset 2 are given in Table 3 and Figure 3.

The performance of the algorithm was identified by studying the machine learning model with the test dataset. Accordingly, 9 features involving dataset 2 was found to be the most effective for KNN and DT accuracy rates compared with the 26 features involving Dataset 1. However, no difference was detected between both datasets for SVM rates. We found that the rem006Fval of the unnecessary data by decreasing the number of features in the dataset significantly increased the accuracy rate of the algorithm compared with the decision tree. As shown in Table 3 and Figure 3, Dataset 2 was the algorithm with the highest accuracy rate of 92.88% compared to the DT algorithm. KNN and SVM were the other algorithms with higher accuracy rates of 87.94% and 86.56%.

The risk of being diagnosed with breast cancer and *BRCA1/2* gene positivity have been studied to be measured with various statistical methods in high-risk individuals for breast cancer. However, the developed statistical models could only identify the possibility of *BRCA1/2* positivity and could not estimate the possibility of *BRCA1/2* positivity with high accuracy [29, 30]. Some of these statistical methods could identify the patient groups from specific ethnic groups and cannot be used in patient groups from different ethnicities. Some of the methods could only estimate the *BRCA1* positivity, and some could only identify the risk for specific age intervals [31–33].

Doncescu and Kabbaj developed a model which could estimate the breast cancer development in accordance with the age of the individual using the machine learning techniques. The age of being diagnosed with breast cancer of the individual is estimated in this modelling based on the *BRCA1/2* gene polymorphisms and familial cancer history. The clusters corresponding to different profiles were identified using an accurate mathematical model for estimating the cluster of the individual in this analysis method. The individuals were evaluated in accordance with this clustering method, and the possibility of getting breast cancer of the individual was found to be higher in the second decade of life using this mathematical method if the healthy individual had 2 relatives diagnosed with breast cancer in their 20s [34].

KhajePasha et al. developed an algorithm which predicted the clinical importance of the *BRCA1/2* single-nucleotide substitution variants with unknown clinical significance using the probabilistic neural network and deep neural network-stacked autoencoder [35]. As a conclusion, intelligent methods were found to have significantly higher estimation rate with no human intervention and were found to be appropriate and applicable methods in resolving various problems when the modelling is performed well and tested with adequate dataset.

Sumitha investigated whether breast cancer could be diagnosed with *BRCA1/2* gene expression analysis using the machine learning algorithms in their study. Researchers investigated the *BRCA1/2* gene expression level using the machine learning algorithms in that study. The algorithms used in the study were the sequential algorithm based on confusion matrix, DCKSVM, and HRBFNN and the prediction algorithms. The HRBFNN algorithm was proven to be effective in analysing the *BRCA1/2* gene expression in the breast cancer dataset in that investigation. Researchers emphasized that it could be made stronger using the other computer-aided algorithms for improving the productivity [22].

The *BRCA1/2* gene test used in the clinical practice is difficult and requires excessive workload and is more expensive and time-consuming compared with the other tests. Longer time of testing causes stress for the patient. In addition, the identification of *BRCA1/2* gene mutation is significantly important for selection of treatment and identification of the risk of the possibility of the secondary cancer. All the above-mentioned algorithms can identify the breast cancer risk in healthy individuals in specific categories and can provide the possibility of carrying *BRCA1/2* gene mutation. However, the rate of the *BRCA1/2* gene mutation carriers is approximately 15%, and 85% of the high-risk patients are unnecessarily tested. Enabling to obtain the clinical results of this expensive, difficult, and long-term taking test and selection of the accurately correct individuals for testing in clinical practice will facilitate the routine practice and reduce the health costs. In addition, selection of *BRCA1/2* gene mutation-negative individuals among the high-risk breast cancer patients is significantly important for reducing the workload and health costs and reducing the stress of the patient and for fast selection of the treatment.

## 4. Conclusions

We evaluated all the clinical, demographic, and genetic features of 2070 patients and developed an algorithm which identifies the *BRCA1/2* gene mutation in a very short time in our study targeting to solve these problems. *BRCA1/2* negativity was identified in 92.88% accuracy with no need for *BRCA1/2* gene testing within minutes in high-risk breast cancer patients using this algorithm and stress of waiting for the test result; time and money loss were prevented. We suggest that this algorithm will be useful in the rapid and accurate performing of the treatment planning of the patients in addition to accelerating the clinical practice.

## Figures and Tables

**Table 1:** A part of raw dataset.

Data number	Diagnosis	Age	FIB	.	.	.	FIO	FIO > 40	BRCA
n0	1	38	0	.	.	.	0	0	0
n1	5	43	0	.	.	.	0	0	0
.	.	.	.	.	.	.	.	.	.
.	.	.	.	.	.	.	.	.	.
n2068	2	48	1	.	.	.	0	0	1
n2069	2	43	1	.	.	.	0	0	1

**Table 2:** Patient and feature information of the datasets.

Datasets	Patient number (*n*)	Feature number (*n*)	Features
Raw dataset	2070	125	Dia, age, sex, CS, PS, HG, MS, NI, ER, PR, HER2, RS, NOBCIF, NOOCIF, NOOTIF, NOTCIF, GR, S, AI, MG, P, LS, M, OC, HI, BRCA etc.
Dataset 1	1460	26	Dia, age, sex, CS, PS, HG, MS, NI, ER, PR, HER2, RS, NOBCIF, NOOCIF, NOOTIF, NOTCIF, GR, S, AI, MG, P, LS, M, OC, HI, BRCA
Dataset 2	1532	9	Dia, age, FIB, SEB, THB, THB < 40, FIO, FIO > 40, BRCA

Dia: diagnosis; CS: clinical stage; PS: pathological stage; HG: histological grades; MS: metastasis status; NI: node involvement; ER: estrogen receptor; PR: progesterone receptor; RS: receptor status; NOBCIF: number of breast cancer in family; NOOCIF: number of ovarian cancer in family; NOOTIF: number of other tumors in family; NOTCIF: number of total cancer in family; FIB: number of first degree relative in breast; SEB: number of second degree relative in breast; THB: number of third degree relative in breast; THB < 40: number of third degree relative in breast under age of 40; FIO: number of first degree relative in ovarian; FIO > 40: number of first degree relative in ovarian above age of 40; GR: geographical regions; S: smoking; AI: alcohol intake; MG: mammography; P: pregnancy; LS: lactation status; M: menopause; OC: oral contraceptive; HI: hormone intake.

**Table 3:** Results of the supervised machine learning algorithms.

Method	Dataset	Number of features (*n*)	Accuracy (%)	Precision (%)	Recall (%)	F1 score (%)
SVM	Dataset 1	26	86.72	75	87	81
	Dataset 2	9	86.56	75	87	80
KNN	Dataset 1	26	85.68	78	86	81
	Dataset 2	9	87.94	86	88	85
DT	Dataset 1	26	78.83	80	79	79
	Dataset 2	9	92.88	93	93	92

SVM: support vector machines; KNN: *k* nearest neighbours; DT: decision tree.

## Data Availability

The data used for the findings of this study are restricted by the Local and Clinical Research Ethics Committee of Istanbul University in order to protect patient privacy.
